# Duck Interleukin-22: Identification and Expression Analysis in *Riemerella anatipestifer* Infection

**DOI:** 10.1155/2021/3862492

**Published:** 2021-11-11

**Authors:** Rochelle A. Flores, Paula Leona T. Cammayo, Binh T. Nguyen, Cherry P. Fernandez-Colorado, Suk Kim, Woo H. Kim, Wongi Min

**Affiliations:** ^1^College of Veterinary Medicine & Institute of Animal Medicine, Gyeongsang National University, Jinju 52828, Republic of Korea; ^2^Department of Veterinary Paraclinical Sciences, College of Veterinary Medicine, University of the Philippines Los Baños College, Laguna 4031, Philippines

## Abstract

*Riemerella anatipestifer* is one of the most devastating pathogens affecting the global duck farms. Infection is involved in secretion of proinflammatory cytokines, including interleukin- (IL-) 17A. During the immune response to infection, IL-22 and IL-17A are often produced concurrently and at high levels in inflamed tissues. Little is known about duck IL-22 (duIL-22) during *R. anatipestifer* infection. We describe the characterization of duIL-22 and its mRNA expression analysis in splenic lymphocytes and macrophages treated with heat-killed *R. anatipestifer* and in the spleens and livers of *R. anatipestifer*-infected ducks. Full-length cDNA of duIL-22 encoded 197 amino acids. The deduced amino acid sequence of duIL-22 shared a 30.4–40.5% similarity with piscine counterparts, 57.4–60.1% with mammalian homologs, and 93.4% similarity to the chicken. Duck IL-22 mRNA expression level was relatively high in the skin of normal ducks. It was increased in mitogen-stimulated splenic lymphocytes and in killed *R. anatipestifer*-activated splenic lymphocytes and macrophages. Compared with healthy ducks, IL-22 transcript expression was significantly upregulated in the livers and spleens on days 1 and 4 postinfection, but not on day 7. IL-17A was significantly increased in the spleens only on day 4 postinfection and in the livers at all time points. When splenic lymphocytes were stimulated with heat-killed *R. anatipestifer*, CD4^+^ cells predominantly produced IL-22 while IL-17A was expressed both by CD4^+^ and CD4^−^ cells. These results suggested that IL-22 and IL-17A are likely expressed in different cell types during *R. anatipestifer* infection.

## 1. Introduction

As the originally designated interleukin- (IL-) 10-related T cell-derived inducible factor (IL-10 TIF), IL-22 is one of the best-studied members of the IL-10 cytokine family [1]. IL-22 is produced by cells of the adaptive and innate immune system, such as natural killer T cells, natural killer cells, *γδ* T lymphocytes, type 3 innate lymphoid cells, macrophages, and activated Th1, Th17, and Th22 cells [2, 3]. IL-22 is an *α*-helical cytokine and functions through engagement to a heterodimer receptor consisting of IL-10 receptor 2 (IL-10R2) and IL-22R1 subunits. IL-10R2 is broadly expressed, while IL-22R1 expression is mostly limited to intestinal and respiratory epithelial cells, keratinocytes, hepatocytes, and kidney, but not immune cells [1, 4, 5]. IL-22 binding to its receptor complex triggers phosphorylation of Jak1 and Tyk2 and activation of the transcription factor signal transducer and activator of transcription 3 pathway and, to a lesser extent, the mitogen-activated protein kinase pathway [4]. IL-22 induces expression of proinflammatory cytokines, including granulocyte colony-stimulating factor, IL-6, and IL-1*β*. It also promotes production of antimicrobial peptides (AMPs) (e.g., *β*-defensin-2 and *β*-defensin-3) and chemokines (CXCL1, CXCL5, and CXCL9) [2, 4, 6].

IL-22 is well-identified and characterized in mammals and in many lower vertebrates, such as fugu [7], zebra fish [8], western clawed frog [9], Atlantic cod and haddock [10], rainbow trout [11], turbot [12], and so-iuy mullet [13]. In avian species, a putative sequence was found in turkey [14] and identified in chickens [15]. In chickens, costimulation of chicken embryo kidney cells with LPS and recombinant IL-22 enhances secretion of proinflammatory cytokines, chemokines, and AMPs [15]. There is little information about the function of IL-22 during the avian immune response.

*Riemerella anatipestifer* is a Gram-negative, nonmotile, and non-spore-forming bacterium belonging to the Flavobacteriaceae family. It induces acute and chronic septicemia manifested by airsacculitis, perihepatitis, fibrinous pericarditis, and meningitis [16]. Clinical disease primarily affects most bird species, including domestic ducks, turkeys, geese, chickens, and other wild birds [16–18]. At least 21 serotypes of *R. anatipestifer* that differ in virulence both between and sometimes within a given serotype have been identified, with less or no significant cross-protection among serotypes. Infection depending on strain-associated virulence causes mortality rate in a 5–75% range [16, 19, 20]. Although *R. anatipestifer* is an infectious disease that presents a significant threat of economic loss in the duck farms over the world [16], very little is known about the mechanisms of protective immunity underlying the pathogenesis of *R. anatipestifer*.

Our comparative expression analyses of immune-associated genes in chickens and ducks found that IL-17A is significantly upregulated during *R. anatipestifer* infection in ducks and in splenic lymphocytes stimulated with heat-killed *R. anatipestifer* [21–23]. IL-22 and IL-17A are cytokines coexpressed by Th17 cells in response to IL-23 [24, 25]. IL-22 and IL-17A exert similar protective function in controlling extracellular bacterial infection by inducing strong mucosal immunity [26] via induction of AMPs, recruitment of neutrophils to the site of bacterial invasion, and enhancement of mucosal barrier maintenance by stimulation of epithelial cell proliferation and tight junction protein production [24, 27, 28]. On the contrary, coexpression of IL-22 and IL-17A has been implicated in the development of proinflammatory processes during inflammatory and autoimmune diseases, such as psoriasis and Crohn's disease [25, 26, 29].

The goal of this study was to identify and analyze gene expression of IL-22 in ducks. We also examined the potential association between IL-22 and IL-17A in *R. antipestifer-*infected ducks. We, here, describe the full-length cDNA encoding duck IL-22 (duIL-22) protein, together with gene expression analyses in normal tissues and mitogen-treated splenic lymphocytes. This study found that IL-22 was upregulated during *R. anatipestifer* infection and was predominantly produced by CD4^+^ cells. IL-17A was produced by CD4^+^ cells and other cell types. These results suggested that there are differences in IL-17A and IL-22 production during *R. anatipestifer* infection.

## 2. Materials and Methods

### 2.1. Animals and Infection

Pekin ducklings were purchased (Joowon ASTA Ducks, Korea). Throughout the experiment, they were kept in wire cages in temperature-controlled conditions and provided *ad libitum* access to water and food. Sixty ducks were injected via the intramuscular route with 5 × 10^7^ CFU of *R. anatipestifer* serotype 7 suspended in 200 *μ*l PBS. On days 1, 4, and 7 after infection, the spleens and livers were harvested from five ducks at each time point for gene expression analysis. Similarly, control birds were injected with 200 *μ*l phosphate-buffered saline (PBS), and the spleens were collected and analyzed time-matched to the infected group. All experiments were maintained in strict accordance with Gyeongsang National University (GNU) *Guide to the Care and Use of Experimental Animals* and were approved by IACUC (GNU-180206-M0009). The bacteria were grown first in sheep blood agar plates (Asan Pharmaceutical, Korea) in a 37°C incubator for 48 h with 5% CO_2_. A single selected colony was inoculated into tryptic soy broth (TSB; Difco, USA) and incubated in a shaking incubator at 37°C until the logarithmic growth phase. Serial dilutions (1 : 10) of the bacterial stock were plated onto sheep blood agar plates to determine the final concentration of the stock to be used as the inoculum.

### 2.2. Molecular Cloning and Sequence Analysis of DuIL-22

Specific primers (Table 1) for reverse transcription-polymerase chain reaction (RT-PCR) were designed from the predicted sequence to obtain a partial sequence of the internal regions of duIL-22 cDNA from duck splenic lymphocytes stimulated with ConA. With a partial sequence information obtained, full-length duIL-22 cDNA was generated by applying 5′/3′ rapid amplification of cDNA ends (RACE) with the specific primers (Table 1), high-fidelity DNA polymerase (Bioneer, Korea), and a 5′/3′ RACE kit (2^nd^ Generation; Roche Applied Sciences, Germany), following the manufacturer's protocol. RT-PCR products were then cloned to TA Vector (RBC, Taiwan) and sequenced (Macrogen, Korea). RT-PCR was performed on a thermocycler (Bio-Rad, Hercules, CA, USA) using 5 min at 95°C; 35 cycles of 1 min at 95°C, 1 min at 55°C, and 1 min at 72°C; and a final 5 min extension at 72°C. The cloned duIL-22 cDNA sequence was submitted to GenBank (accession number MT360382).

The cloned sequence was analyzed for similarity and percent identity with other known homologue sequences using EMBOSS Needle pairwise alignment (http://www.ebi.ac.uk/Tools/psa/emboss_needle). Protein identification was performed using the Expert Protein Analysis System (ExPASy; http://www.expasy.org/tools/). Amino acid sequence comparison was performed using Clustal Omega multiple sequence alignment (http://www.ebi.ac.uk/Tools/msa/clustalo/). The signal peptide sequence, N-glycosylation site, and the theoretical pI value and molecular weight were predicted using the SignalP 4.1 Server (http://www.cbs.dtu.dk/services/SignalP), NetNGlyc 1.0 Server (http://www.cbs.dtu.dk/services/NetNGlyc/), and Compute pI/Mw (http://www.expasy.org/compute_pi), respectively. A phylogenetic tree was generated based on multiple alignments of the protein sequences of IL-10 cytokine family members using the CLUSTAL X Multiple Sequence Alignment Program and neighbor-joining method in the MEGA7 program with a bootstrap value of 10,000 for the evolutionary analysis [30].

### 2.3. Cell Culture

Lymphocytes and macrophages were obtained from the spleens of two-week-old healthy ducks [31, 32]. The cells were grown in Dulbecco's modified Eagle's medium (DMEM) (Gibco Life Technologies, USA) containing 1% penicillin-streptomycin (10,000 units/ml) and 10% fetal bovine serum in a 41°C incubator with 5% CO_2_. A concentration of 5 × 10^6^ cells/ml splenic lymphocytes was stimulated using either 10 *μ*g/ml concanavalin A (ConA; Amersham Bioscience, Sweden), 25 *μ*g/ml polyinosinic:polycytidylic acid (poly I:C; Sigma-Aldrich, Germany), 10 *μ*g/ml lipopolysaccharide (LPS from *Escherichia coli*, 0111:b4; Sigma-Aldrich), or heat-killed *R. anatipestifer* (1 × 10^6^ CFU/ml) during 4, 8, or 24 h. Primary macrophage was seeded (5 × 10^6^ cells/well) and stimulated with *R. anatipestifer* killed by boiling (1 × 10^6^ CFU/ml) during 4, 8, or 24 h. The *R. anatipestifer* killed by boiling was obtained by placing at 100°C for 5 min in a water bath. Following the same protocol, COS-7 cells were cultured in a 37°C incubator with 5% CO_2_.

### 2.4. Construction of Plasmid and Transfection

A duIL-22 sequence with MYC-tag (duIL-22-MYC construct) was amplified from ConA-treated splenic lymphocytes using RT-PCR and the specific primers: forward 5′-GATCAAGCTTATGGCCTCCCTGCAGACCTT-3′ and reverse 5′-GATCGAATTCTCACAGATCCTCTTCTGAGATGAGTTTTTGTTCGTTTTTCTTCTTATTCCCTC-3′. A single solid underline indicated the restriction enzyme sites, *HindIII* and *EcoRI*; a dashed underline indicated the MYC-expressing sequence. Synthesized full-length duIL-22 was purified using a FavorPrep GEL/PCR Purification Mini Kit (Favorgen, Taiwan), following the manufacturer's instructions. PCR products digested with *Hind*III and *Eco*RI were ligated into pcDNA 3.1+ vector (Invitrogen, Waltham, MA, USA), transformed into DH5*α* competent cells (RBC), and then confirmed by sequencing (Macrogen). COS-7 cells were transfected with 10 *μ*g of duIL-22-MYC construct using FuGene 6 transfection reagent (Promega, Madison, WI, USA), following the manufacturer's protocol. Mock transfection was performed using the empty pcDNA 3.1+ vector. Transfected cells were cultured in the serum-free medium, Opti-MEM® I (Gibco Life Technologies, USA), at 37°C for 48 h in 5% CO_2_ conditions.

### 2.5. Western Blotting

As previously described [23], cell lysis, sample preparation, SDS-PAGE, and Western blot analysis were carried out. Blotted membranes were incubated with monoclonal anti-MYC mouse antibody (Cell Signaling Technology, Danvers, MA, USA) and horseradish peroxidase-conjugated goat anti-mouse IgG antibody (Promega). The membranes were then washed, incubated with EZ West Lumi plus (ATTO, Japan), and were visualized using a ChemiDoc Imaging System (Bio-Rad, USA).

### 2.6. Splenic CD4^+^ Cell Isolation

Single-cell suspensions were collected from the spleens of two-week-old normal ducks [32]. The lymphocyte was isolated by using Ficoll-Paque PLUS ((GE Healthcare Bio-Sciences, Sweden)). After isolation, the cells were treated using *R. anatipestifer* killed by boiling (1 × 10^6^ CFU/ml) and were sorted by using an anti-PE multisort kit (Miltenyi Biotech, Auburn, CA), following the manufacturer's protocol. Briefly, the cells were incubated with PE-conjugated anti-chicken CD4 and MACS buffer (PBS buffer supplemented with 0.5% BSA and 2 mM EDTA). After incubation, unbound antibodies were removed using centrifugation, and the pellet was resuspended and incubated with anti-PE multisort beads and MACS buffer. Unbound beads were removed using centrifugation, and the CD4^+^ cells were collected using positive selection in a LS column (Miltenyi Biotech, USA).

### 2.7. Quantitative Real-Time RT-PCR (qRT-PCR)

Total RNA from cells and tissues of *R. anatipestifer*-infected ducks and the time-matched untreated/uninfected control ducks were isolated using RiboEx (GeneAll, Korea). They were purified using an RNeasy mini kit (Qiagen, Germany). RNA was subjected to reverse transcriptase using a QuantiTect reverse transcription kit (Qiagen, Germany) with a random hexamer primer. cDNA was subjected to qRT-PCR analysis with SYBR Green (Bioneer, Korea) and gene-specific primers (Table 1) by using a CFX 96 real-time PCR system (Bio-Rad). Relative expression levels of each transcript were quantified using the ^−ΔΔ^CT method and normalized with a reference gene *β*-actin.

### 2.8. Statistical Analysis

The measurements were represented as the mean ± standard error (SE) of the mean values from at least two independent experiments. The data were analyzed with InStat® software (GraphPad, USA) and were tested using Student's *t-*tests or one-way analysis of variance (ANOVA), followed by Dunnett's multiple comparison tests. A *P* value < 0.05 was considered to be a statistically significant result.

## 3. Results

### 3.1. Cloning and Characterization of DuIL-22 cDNA

**Figure 1:**
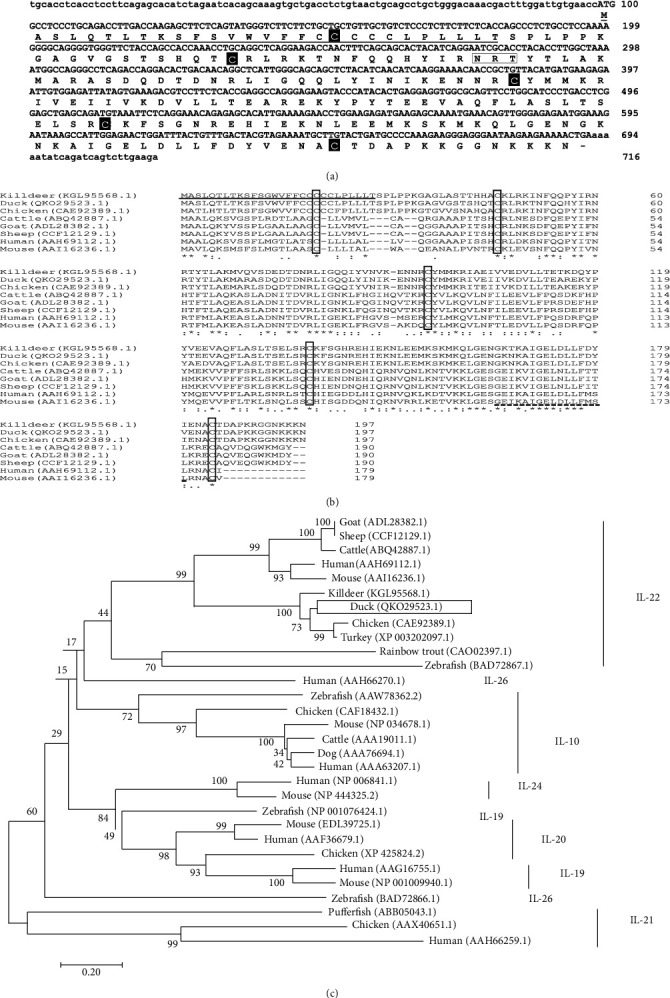
Molecular characterization and genetic analysis of duck IL-22 cDNA. (a) Sequences of nucleotide and deduced amino acid of duck IL-22. The underline indicated the predicted signal peptide region, black boxes indicated the conserved cysteine residues, and white box indicated the putative *N-*linked glycosylation area. (b) Multiple alignment of amino acid sequence of avian and mammalian IL-22. Clustal Omega software was used for multiple alignments. The identical residues among sequences were indicated with asterisks (∗). The underline indicated the signal peptide of duck IL-22, white boxes indicated the conserved cysteine residues between species, and the conserved IL-10 family motif is underlined using a dashed line. GenBank accession numbers used in the comparison are written after the species names. (c) Phylogenetic tree indicating the relationships between IL-22 amino acid sequences and other known IL-10 family members. The tree was made using the neighbor-joining method (MEGA version 7 program) and amino acid multiple alignments. The node values indicated percentage bootstrap confidence levels obtained from ten thousand replicates. The sequence accession numbers are written after the species name. The white box indicated duck IL-22.

The full-length cDNA of duIL-22 was cloned from ConA-stimulated splenic lymphocytes. It consisted of a 716 bp sequence with a 594 bp open reading frame (ORF) to encode 197 amino acids (aa) with a predicted molecular weight of 22.5 kDa (nonglycosylated) and an isoelectric point of 9.18. The protein was predicted to have a 28-aa signal peptide sequence, a 169-aa mature peptide, and one putative N-linked glycosylation area (Asn-X-Ser/Thr) at position 60 (Figure 1(a)). Multiple sequence alignment of duIL-22 to its homologs on other vertebrates revealed five conserved cysteine residues and the presence of a relatively well conserved IL-10 family motif (Figure 1(b)). Comparison of the duIL-22 nucleotide sequence to its piscine, mammalian, and chicken IL-22 homologs revealed identities of 9.2–35.6%, 35.4–55.3%, and 66.6%, respectively. Analysis of amino acids revealed that duIL-22 shared a 30.4–40.5% with piscine counterparts, 57.4–60.1% with mammalian homologs, and 93.4% similarity to chickens (Table 2). The relationships between the identified duIL-22 and other members of the IL-10 family were further investigated by constructing a phylogenetic tree with the neighbor-joining method in the MEGA7 program. Analysis of the tree revealed that the duIL-22 protein was more closely related to its bird counterparts than to its piscine and mammalian counterparts. Likewise, IL-22 formed a distinct branch from other members of the IL-10 cytokine family (Figure 1(c)).

### 3.2. Molecular Weight and Expression Analysis of DuIL-22 Transcript

**Figure 2:**
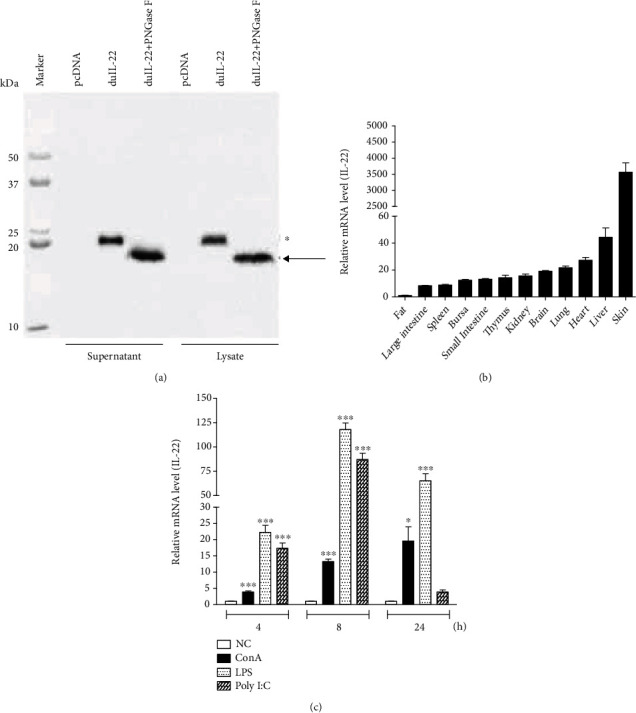
Molecular weight of duck IL-22 and its expression in normal tissues and stimulated splenic lymphocytes. (a) Western blot analysis to determine molecular weight of duck IL-22 protein from COS-7 cells transfected with duIL-22-MYC construct. Supernatants and cell lysates of COS-7 cells were obtained at 48 h after transfection and deglycosylated using PNGase F (100 U peptide-*N*-glycosidase F) at 37°C for 1 h. Supernatants and cell lysates under reducing conditions were separated using SDS-PAGE. An anti-MYC antibody was used to detect the specific bands of duck IL-22. The asterisk (∗) and arrow indicated the normal bands and the deglycosylated bands of duck IL-22, respectively. (b) Distribution of IL-22 transcripts in normal healthy duck tissues. Total RNA was obtained from tissues of two-week-old normal ducks (*n* = 5) and used to qRT-PCR analysis. Expression levels of *β*-actin were used for normalization of IL-22 expression levels, which were calibrated to the lowest expression level detected. Results are presented as the mean ± SE values from 2 independent experiments. (c) Duck IL-22 expression levels in mitogen-treated splenic lymphocytes. The lymphocytes were obtained from two-week-old normal ducks using Ficoll density gradient centrifugation. They were activated using 25 *μ*g/ml poly I:C, 10 *μ*g/m LPS, or 10 *μ*g/ml ConA for the indicated times. Expression levels of *β*-actin gene were used for normalization of IL-22 expression level, which were calibrated with untreated cultured splenic lymphocytes (NC). Results are presented as the mean ± SE values from 2 independent experiments performed in triplicate. ^∗^*P* < 0.05 and ^∗∗∗^*P* < 0.001.

Western blot analyses identified a 22.8 kDa and a 19 kDa protein in the supernatants and lysates of COS-7 cells which are transfected with duIL-22-MYC construct. The 22.8 kDa protein (calculated molecular weight of 22.5 kDa; asterisk in Figure 2(a)) presented the *N*-linked glycosylated form of duIL-22 in cell supernatants and lysates (Figure 2(a)). The duIL-22 expression was monitored for various normal healthy tissues (Figure 2(b)) and mitogen-treated splenic lymphocytes (Figure 2(c)) using qRT-PCR. IL-22 was detectable for all the tested tissues; duck fat had the lowest expression level. Compared with expression in fat, IL-22 mRNA expression levels were high in the skin; moderate levels were detected in the liver, heart, lungs, and brain. Low levels of IL-22 transcripts were found in the intestines and lymphoid organs (Figure 2(b)). DuIL-22 mRNA transcripts on mitogen-stimulated lymphocytes such as LPS, PolyI:C, and ConA were generally upregulated at all times, compared with unstimulated cultured splenic lymphocytes (Figure 2(c)).

### 3.3. IL-22 and IL-17A Are Upregulated during *R. anatipestifer* Stimulation and Infection

**Figure 3:**
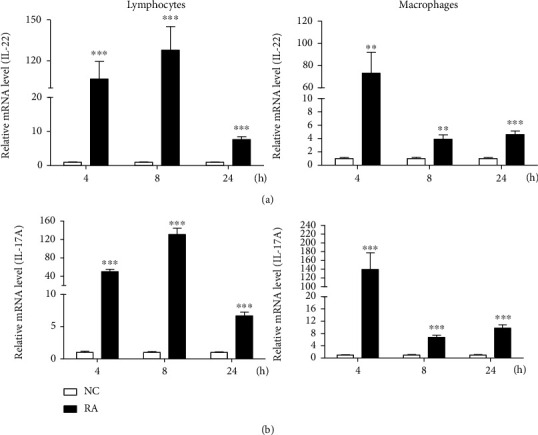
mRNA expression profiles of duck IL-22 in splenic lymphocytes and macrophages. Lymphocytes and macrophages were collected and isolated from 2-week-old healthy ducks and treated with heat-inactivated *R. anatipestifer* serotype 7 for the indicated times. RNA was isolated and samples were applied to qRT-PCR analysis. The transcript expression levels of IL-22 (a) and IL-17A (b) were normalized to expression levels of *β*-actin used as a reference gene and presented relative to the expression levels of untreated/cultured lymphocytes and macrophages (NC). Results are presented as the mean ± SE values from 3 independent experiments performed in triplicate. ^∗∗^*P* < 0.01 and ^∗∗∗^*P* < 0.001. RA: *R. anatipestifer*-stimulated lymphocytes.

**Figure 4:**
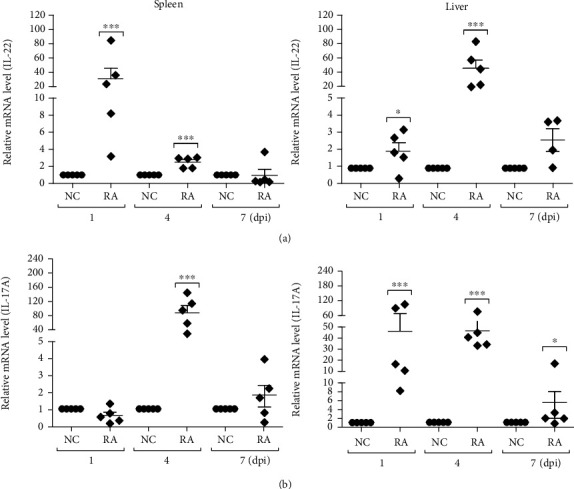
Expression profiles of IL-22 in ducks infected with *R. anatipestifer*. Expression levels of IL-22 (a) and IL-17A (b) transcripts from the spleens and livers of ducks infected intramuscularly with 5 × 10^7^ CFUs of *R. anatipestifer* serotype 7. The spleens and livers of 2-week-old healthy ducks were aseptically sampled on days 1, 4, and 7 postinfection (dpi). Tissue samples were pooled, and total RNA was isolated for qRT-PCR. Gene expression levels were normalized with *β*-actin used as a reference gene and presented relative to the expression levels of uninfected and healthy controls (NC). These results represented one representative of 2 independent experiments. Results are presented as the mean ± SE values (*n* = 5). ^∗^*P* < 0.05 and ^∗∗∗^*P* < 0.001. dpi: days postinfection; RA: *R. anatipestifer*-infected ducks.

Gene expression analyses of Th17-associated cytokines from our previous studies showed that IL-17A and IL-17F expression was increased in the spleens and livers of *R. anatipestifer-*infected ducks and in splenic lymphocytes treated with *R. anatipestifer* killed by boiling [22, 33]. Both IL-22 and IL-17A promoted induction of AMP; they are implicated together in the inflammatory processes involved in cases of psoriasis, inflammatory bowel diseases, and rheumatoid arthritis [24, 34, 35]. These findings suggested that both cytokines have similar roles. Thus, the mRNA expression patterns of IL-17A and IL-22 were monitored in duck splenic macrophages and lymphocytes activated with heat-inactivated *R. anatipestifer* (Figure 3) and in *R. anatipestifer*-infected ducks (Figure 4). Compared with the untreated cultured controls, IL-17A and IL-22 expression was significantly increased at all time points in the splenic lymphocytes and macrophages treated with heat-inactivated *R. anatipestifer*. Expression levels in the stimulated splenic lymphocytes showed changes of 7.7–127.8-fold and 6.7–131.2-fold for IL-22 and IL-17A, respectively. Similarly, when compared to their unstimulated culture controls, we found an upregulated expression of a 3.9–73.2-fold change for IL-22 and a 6.7–139.5-fold change for IL-17A on splenic macrophages stimulated with heat-inactivated *R. anatipestifer*. These findings suggested that duck IL-22 and IL-17A were upregulated *in vitro* during stimulation with *R. anatipestifer*.

As both cytokines were upregulated in the splenic macrophages and lymphocytes stimulated with heat-inactivated *R. anatipestifer*, the expression profiles of these cytokines were monitored in the spleens and livers of infected ducks at days 1, 4, and 7 postinfection. Interestingly, compared with the healthy controls, IL-22 was significantly upregulated in the spleens and livers of ducks infected with *R. anatipestifer* at days 1 and 4, but not on day 7. IL-17A was detected at high expression levels in the livers of infected ducks at all time points, but expression was only significantly upregulated at day 4 in the spleens of infected ducks with a 105-fold change compared with healthy control animals. These results suggested that IL-22 and IL-17A were regulated differently during *R. anatipestifer* infection.

### 3.4. IL-22 Is Mainly Produced by CD4^+^ Cells

**Figure 5:**
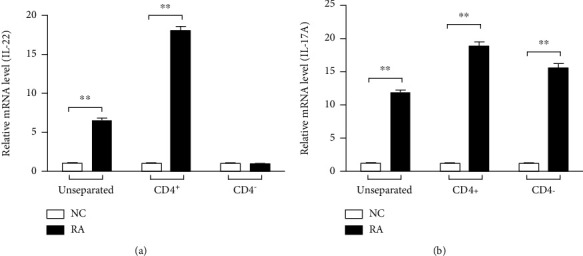
IL-22 is produced primarily by CD4^+^ cells during *R. anatipestifer* infection, but not IL-17. The spleens were collected from 2-week-old healthy ducks. Spleen cells were isolated, cultured, and stimulated with heat-killed *R. anatipestifer* serotype 7. After 24 h, cells were collected, labelled, and separated following incubation in MACS buffer. Total RNA was obtained, and qRT-PCR analysis was performed. Gene expression levels of IL-22 (a) and IL-17A (b) transcripts were normalized with *β*-actin used as a reference gene and calibrated with the expression levels of untreated cultured spleen cells (NC). These results represented one representative of 2 independent experiments. Results are presented as the mean ± SE values performed in triplicate. ^∗∗^*P* < 0.01. RA: splenic lymphocytes stimulated with heat-inactivated *R. anatipestifer*.

IL-22 and IL-17A are cytokines coexpressed by Th17 cells in response to IL-23 [24, 25]. Recently, duck IL-23p19 was found to be unimportant in the IL-17A response during the early stages of *R. anatipestifer* infection in ducks [23]. Thus, it would be interesting to know what cells might be involved in the production of IL-22 during the infection, and more so if IL-22 is somewhat produced in the same manner or maybe regulated differently with IL-17A during *R. anatipestifer* infection. Splenic lymphocytes of ducks were activated with heat-inactivated *R. anatipestifer*, and CD4^+^ cells were separated. The CD4^+^ predominantly produced IL-22, while IL-17A was expressed both by CD4^+^ and other cells of the spleen (Figure 5).

## 4. Discussion

Clinical *R. anatipestifer* infection in ducks is considered one of the most economically critical diseases in the duck farms. In duck populations, infection can result in a mortality rate as high as 75%, depending on strain virulence [16, 20]. Our previous studies are associated with upregulated expression of IL-17A and IL-17F mRNA in infected ducks and in activated splenic lymphocytes [22, 33]. The association of IL-17 with infection is further confirmed when increased survival rates and decreased bacterial burdens in ducks infected with *R. anatipestifer* occur after expression levels of IL-17A and IL-17F cytokines are downregulated using berberine treatment [33]. IL-17A is the signature cytokine of Th17 cells and is often associated with various autoimmune and proinflammatory diseases, such as organ-specific inflammation [36] and psoriasis [37]. Th17 cells also express IL-22. IL-22 is a novel IL-10 family member that can induce production of AMPs (*β*-defensins and S100 proteins) [35] and acute phase proteins (serum amyloid A and LPS-binding protein) [38]. Similar to IL-17A, IL-22 is linked to the pathogenesis of various proinflammatory processes. Therefore, this study was performed to investigate whether there was a relationship between IL-22 and IL-17A, or whether they were regulated differently during *R. anatipestifer* infection. To answer this question, duIL-22 was first identified from mitogen (ConA)-treated splenic lymphocytes. We then characterized its molecular features and expression profiles of transcripts in normal tissues of ducks, in mitogen-stimulated cells, and in the spleens and livers of *R. anatipestifer*-infected ducks. We also monitored expression levels of IL-22 and IL-17A in CD4^+^ and CD4^−^ cells separated from heat-killed *R. anatipestifer*-stimulated splenic lymphocytes.

This study was the first to perform molecular cloning and characterization of IL-22 in ducks. Sequence alignment of duIL-22 protein with the deduced amino acid sequences of IL-22 homologs from mammalian and avian species revealed conserved regions, including the IL-10 family signature (G-X2-KA-X2-[D, E]-X-D[ILV]-[FLY]-[FILMV]-X2-[ILMV][EKQR]) [13]. Five cysteine residues were also conserved between mammalian and avian species, compared with their piscine counterparts, who had three or four conserved cysteine residues [10, 13]. Comparison of nucleotide sequence and amino acid sequence of duIL-22 revealed higher identities and similarities to the poultry species, compared with their piscine and mammalian counterparts. Phylogenetic tree analysis of the IL-10 family found that duIL-22 was more closely related to its poultry counterparts than its mammalian or piscine homologs. Moreover, IL-22 was generally more evolutionarily closer to IL-10 and human IL-26 cytokines (Figure 1(c)).

Sequence analysis revealed that duIL-22 contained a 594 bp ORF predicted to encode 197 amino acids with a signal peptide (amino acid 1-28) and a single *N*-linked glycosylation site. A one putative N-glycosylation area in duck IL-22 protein is consistent with the presence of a single putative *N*-glycosylation area in its chicken counterpart. However, mouse and human IL-22 have three sites and rainbow trout have no putative glycosylation sites [11, 15]. Interestingly, when supernatants and lysates of COS-7 cells expressing duIL-22-MYC construct were treated with PNGase-F, Western blot analysis revealed a 19 kDa protein on the supernatants and lysates, whereas a 22.8 kDa protein was detected on the untreated supernatants and lysates. This result suggested that duIL-22 had one *N-*linked glycosylation (Figure 2(a)).

IL-22 tissue distribution experiments found that it was expressed in a wide variety of tissues, primarily in the skin, followed by the liver, heart, lung, brain, and kidney. This result was likely because IL-22R is expressed on stromal and epithelial cells of these tissues [1]. This result differed from distribution in the chicken, in which high expression is found in the thymus, spleen, and intestine (jejunum and ileum) [15]; duIL-22 expression levels were relatively low in these tissues (Figure 2(b)). DuIL-22 distribution was also different from IL-22 distribution in teleost species, in which IL-22 is highly expressed in the intestines, tail fins, gills, and gonads [8, 10, 11]. Although primarily produced by lymphoid tissues, IL-22 is also produced in nonlymphoid sources, such as macrophages in the lungs of humans and mice, in response to lung injury. Experimental models found that IL-22 is produced in neutrophils during colitis and in fibroblasts during rheumatoid arthritis [1].

Expression of duIL-22 was significantly upregulated in mitogen-stimulated splenic lymphocytes at all time points. These results were consistent with results indicating that IL-22 expression is induced in rainbow trout splenocyte primary cultures stimulated with phorbol myristate acetate [11], a potent enhancing factor for T cell colony growth [39]. IL-22 expression is induced in turbot head kidney, spleen, and intestine cells stimulated with phorbol myristate acetate and phytohemagglutinin [12]. Similarly, expression of IL-22 and IL-17A transcripts in splenic lymphocytes and macrophages was stimulated with heat-killed *R. anatipestifer* at all times. Thus, to study a possible link between IL-22 and IL-17A in *R. anatipestifer* infection, ducks were injected with *R. anatipestifer* via the intramuscular route, and the spleens and livers were collected and analyzed at days 1, 4, and 7 postinfection. Unlike our *in vitro* findings that both cytokines which were expressed at all times are expressed with a similar pattern, only IL-17A was significantly induced in the liver at all time points; IL-22 was induced on days 1 and 4, but not on day 7. In the spleen, IL-22 was significantly expressed on days 1 and 4 after infection, while significant IL-17A expression was found only day 4. Although, to a certain extent, IL-22 and IL-17A expression was overlapped in the spleens of ducks infected with *R. anatipestifer*, these results implied a possibility that IL-22 and IL-17A are expressed in different cell types of *R. anatipestifer*-infected ducks. It is interesting to note that distinct expression of IL-17A and IL-23p19, unlike in vitro expression patterns analyzed with *R. anatipestifer*-treated splenic lymphocytes, was identified *in vivo* analyzed with the spleens of ducks infected with *R. anatipestifer* [23]. Differential expression of IL-22 and IL-17A was confirmed in peritoneal lavage fluids of *Salmonella* Enteritidis-infected mice; IL-22 was induced on day 1, but not on day 14, whereas IL-17A was induced on day 14, but not on day 1 [40]. Thus, CD4^+^ cell expression levels were analyzed for further insight on IL-22 and IL-17A production during *R. anatipestifer* infection. IL-22 was detected at significant levels on CD4^+^ cells, while IL-17A was present on CD4^+^ cells and other splenocytes. These findings suggested that IL-22 and IL-17A were expressed in different cell types during *R. anatipestife*r infection.

In conclusion, duIL-22 was cloned to report IL-22 expression profiles on duck tissues and immune cells. Furthermore, we aimed to determine whether there was a connection between IL-22 and IL-17A during *R. anatipestifer* infection because both cytokines are coexpressed by certain cell subsets for protection or during disease progression. Unlike the *in vitro* results, we found that IL-22 and IL-17A were not expressed in the same manner, especially in the spleens of infected ducks. Moreover, while CD4^+^ cells expressed both IL-22 and IL-17A, IL-22 was primarily produced only from CD4^+^ cells. Further investigation is needed to accurately identify subsets of cells that express IL-22.

## Figures and Tables

**Table 1:** Primers used for cloning and expression analysis.

Target genes	Purpose	Orientation and sequence (5′-3′)	References
DuIL-22	3′ RACE	(For) TACCAGCCACCAAACCTGCA	This study
	5′ RACE	(Rev) AATCCAGTTCTCCAATGGC	
	RT-PCR	(For) CTGCCTCCAAAAGGGGCAG	This study
		(Rev) TCAGGGATGCCAGGAACTGC	
	qRT-PCR	(For) CCAGCCACCAAACCTGCAG	This study
		(Rev) GAGCCTGTTGTCAGTGTCC	
DuIL-17A	qRT-PCR	(For) ATGTCTCCAACCCTTCGT	Kim et al. (2015)
		(Rev) CCGTATCACCTTCCCGTA	
*β*-Actin	qRT-PCR	(For) GCTATGTCGCCCTGGATTTC	Liu et al. (2012)
		(Rev) CACAGGACTCCATACCCAAGAA	

RT-PCR: reverse transcription-polymerase chain reaction; qRT-PCR: quantitative real-time polymerase chain reaction; RACE: rapid amplification of cDNA ends; DuIL-22: duck interleukin-22.

**Table 2:** Duck IL-22 percent identity and similarity with IL-22 sequences of other vertebrates.

Species	Duck IL-22				
	Nucleotide		Protein		
	Identity	GenBank Acc. No.	Identity	Similarity	GenBank Acc. No.
Human	55.3	BC069112.1	41.1	59.9	AAH69112.1
Mouse	50.4	BC116235.2	38.3	57.7	AAI16236.1
Sheep	43.9	HE617662.1	36.4	60.1	CCF12129.1
Cattle	35.4	EF560596.1	34.9	57.4	ABQ42887.1
Rainbow trout	35.6	AM748537.1	23.8	40.5	CAO02397.1
Zebrafish	9.2	AB194274.1	19.1	30.3	BAD72867.1
Chicken	66.6	AJ617782.1	86.3	93.4	CAE92389.1

## Data Availability

Data will be available from authors on request.
